# Chloro-Anæmia Treated with Bean of St. Ignatius

**Published:** 1860-03

**Authors:** 

**Affiliations:** Würzburg


*Chloro-Anæmia treated with Bean of Saint Ignatius.* By Dr. Eisenmann, of Würzburg.

Chlorosis is a disease developing itself particularly in medical constitutions, predisposed to nervous affections. It prefers the sex which has the marked predisposition for neuroses, and develops itself at the period of life when all kinds of neuroses are very frequent. Its *début* is marked by the appearance of nervous phenomena, whilst the blood presents not the least alteration, and during its entire persistence numerous nervous phenomena are observed. Any particular alteration of the blood may be wanting, even in cases where the disease is completely developed. It is cured by the use of therapeutic means that exercise a special action on the spinal cord. These considerations authorize me, even force me, to conclude that chlorosis is primarily a nervous affection, and that the alteration of the blood is only a secondary phenomenon, resulting from morbid innervation.

This mode of considering the nature of chloro-anæmia is not an idle theory. It is practically useful, since it leads to the discovery of substances which shall have special curative properties in this disease. I select among these such as contain strychnia and brucia. The first patient on whom I employed this treatment was a strong, robust woman, the wife of a miller, aged about thirty, who said she had chlorosis for eight years, and who had been treated by all the physicians of the neighborhood, without any permanent result. She presented all the symptoms of chloro-anæmia, together with œdema of the lower extremities, and also a somewhat considerable effusion in the abdominal cavity. I gave her, twice a day, from 10 to 15 drops of the tincture of the St. Ignatius bean. Under the sole influence of this medicament all the morbid phenomena, including the œdema of the legs and the abdominal effusion, disappeared in eight weeks.

Soon after I was called to see two young girls, 15½ and 16 years of age. They were frail and delicate; their complexion was florid, clear, and very delicate ; all the symptoms announced that they were affected with chloro-anæmia. A physician, previously consulted, had prescribed ferruginous preparations, until the very sensitive stomachs of the patients could no longer bear them. I

gave them, twice a day, six drops of tincture of St. Ignatius beans, recommending an increase of the dose one drop every three days. At the end of four weeks they were cured; it is true that, in their case, the disease had not made any considerable progress. In some other cases I employed the same agent; and my friend, Dr. Seligsberg, at Kronach, has also experimented with it, and our experiments have fully satisfied our expectations. Being thus convinced of the curative powers of the bean of St. Ignatius in chlorosis, I desired to see if, associated with ferruginous preparations, it would not produce a cure more speedily than when employed alone; and as, in most cases, there is also an obstinate constipation, I added rhubarb to the two substances. The following is my formula:

| | |
|---|---|
| R.—Pulv. Fab. Sanct. Ignatii, | gramme 0.06 |
| Ferri Lact., | " 0.18 |
| Pulv. Rhei, | " 0.18 |
| Misce. | |

Take two powders a day; with this, nutrition and tonic regimen, and exercise in open air. This treatment has always succeeded with me since 1846, except a case in 1852, which proved rebellious under all treatments. In cases where the digestive organs will not bear the iron, I begin by administering the Ignatia alone, and only add the lactate of iron, and afterwards iron in substance, and rhubarb when the sensitiveness has passed away. My formula is so much the more useful, since it overcomes the obstinate constipation which so often accompanies chloro-anæmia. All my friends who have used this treatment in their practice, have noticed that they produced cures much more rapidly than with iron preparations alone, and have found it efficacious when these have failed.—*Bulletin de Thérapeutique.* L. H. S.

